# High *in vitro* activity of the novel antimicrobial gepotidacin against *Neisseria gonorrhoeae* isolates in eight WHO Enhanced Gonococcal Antimicrobial Surveillance Programme countries in three WHO regions, 2021–24

**DOI:** 10.1093/jac/dkaf452

**Published:** 2025-12-16

**Authors:** Josefine Ahlstrand, Ismael Maatouk, Le Huu Doanh, Natnaree Girdthep, Daniel Golparian, Lon Say Heng, Irving Hoffman, Manuel C Jamoralin, Francis Kakooza, Rossaphorn Kittiyaowamarn, Peter Kyambadde, Pham Thi Lan, Venessa Maseko, Mitch Matoga, Etienne Müller, Hao Trong Nguyen, Thuy Thi Phan Nguyen, Vichea Ouk, Vivi Setiawaty, Sonia B Sia, Verawati Sulaiman, Mot Virak, Van Thi Thuy Nguyen, Teodora Wi, Susanne Jacobsson, Magnus Unemo, the WHO EGASP Gepotidacin Study Group, Lon Say Heng, Vichea Ouk, Mot Virak, Phal Kun Mom, Serongkea Deng, Vivi Setiawaty, Endang Lukitosari, Nurhalina Afriana, Verawati Sulaiman, Teguh Hartono, Maria Laurensia, Ni Luh Putu Pitawati, Mitch Matoga, Irving Hoffman, Robert Krysiak, Sonia Sia, Manuel C Jamoralin, Marietta Lagrada, June Gayeta, Jaywardeen Abad, Venessa Maseko, Etienne Müller, Lindy Gumede, Duduzile Valashiya, Rossaphorn Kittiyaowamarn, Natnaree Girdthep, Porntip Paopang, Pongsathorn Sangprasert, Thitima Cherdtrakulkiat, Jaray Tongtoyai, Francis Kakooza, Peter Kyambadde, Emmanuel Mande, Martha Nakasi, Dickson Tabajjwa, Le Huu Doanh, Pham Thi Lan, Pham Quynh Hoa, Pham Dieu Hoa, Thuy Thi Phan Nguyen, Hao Trong Nguyen, Nhi Thi Uyen Pham, Phuong Thi Thanh Nguyen, Nguyen Thi Thuy Van, Monica Lahra, Teodora Wi, Ismael Maatouk, Phiona Vumbugwa, Magnus Unemo, Daniel Golparian, Susanne Jacobsson, Daniel Schröder

**Affiliations:** Department of Laboratory Medicine, Faculty of Medicine and Health, WHO Collaborating Centre for Gonorrhoea and Other Sexually Transmitted Infections, National Reference Laboratory for Sexually Transmitted Infections, Örebro University, Örebro, Sweden; Global HIV, Hepatitis and STI Programmes, WHO, Geneva, Switzerland; National Hospital of Dermatology and Venereology and Hanoi Medical University, Hanoi, Vietnam; Bangrak STIs Center, Division of AIDS and STIs, Department of Disease Control, Thailand Ministry of Public Health, Bangkok, Thailand; Department of Laboratory Medicine, Faculty of Medicine and Health, WHO Collaborating Centre for Gonorrhoea and Other Sexually Transmitted Infections, National Reference Laboratory for Sexually Transmitted Infections, Örebro University, Örebro, Sweden; National Center for HIV/AIDS, Dermatology and Sexually Transmitted Diseases, Phnom Penh, Cambodia; UNC Project Malawi, Lilongwe, Malawi; Department of Health, Research Institute for Tropical Medicine, Manila, the Philippines; Infectious Diseases Institute, Makerere University College of Health Sciences, Kampala, Uganda; Bangrak STIs Center, Division of AIDS and STIs, Department of Disease Control, Thailand Ministry of Public Health, Bangkok, Thailand; Sexually Transmitted Infections Program, Ministry of Health, Kampala, Uganda; National Hospital of Dermatology and Venereology and Hanoi Medical University, Hanoi, Vietnam; Centre for HIV and STIs, National Institute for Communicable Diseases, National Health Laboratory Service, Johannesburg, South Africa; UNC Project Malawi, Lilongwe, Malawi; Centre for HIV and STIs, National Institute for Communicable Diseases, National Health Laboratory Service, Johannesburg, South Africa; Ho Chi Minh City Hospital of Dermatology and Venereology, Ho Chi Minh City, Vietnam; Ho Chi Minh City Hospital of Dermatology and Venereology, Ho Chi Minh City, Vietnam; Laboratory of the National Institute of Public Health, Phnom Penh, Cambodia; Sulianti Saroso Infectious Disease Hospital, Jakarta, Indonesia; Department of Health, Research Institute for Tropical Medicine, Manila, the Philippines; Sulianti Saroso Infectious Disease Hospital, Jakarta, Indonesia; National Center for HIV/AIDS, Dermatology and Sexually Transmitted Diseases, Phnom Penh, Cambodia; WHO Country Office, Hanoi, Vietnam; Global HIV, Hepatitis and STI Programmes, WHO, Geneva, Switzerland; Department of Laboratory Medicine, Faculty of Medicine and Health, WHO Collaborating Centre for Gonorrhoea and Other Sexually Transmitted Infections, National Reference Laboratory for Sexually Transmitted Infections, Örebro University, Örebro, Sweden; Department of Laboratory Medicine, Faculty of Medicine and Health, WHO Collaborating Centre for Gonorrhoea and Other Sexually Transmitted Infections, National Reference Laboratory for Sexually Transmitted Infections, Örebro University, Örebro, Sweden; Institute for Global Health, University College London (UCL), London, UK

## Abstract

**Objectives:**

The spread of ceftriaxone-resistant *Neisseria gonorrhoeae* is threatening the last option for gonorrhoea treatment, ceftriaxone. Gepotidacin, the first-in-class triazaacenaphthylene bacterial topoisomerase type IIA inhibitor, recently showed non-inferiority compared to ceftriaxone-azithromycin for treatment of uncomplicated urogenital gonorrhoea in a Phase 3 randomized controlled trial. We evaluated the *in vitro* susceptibility to gepotidacin in clinical gonococcal isolates (*n* = 2912), including 125 (4.3%) ceftriaxone-resistant isolates, collected 2021–24 in eight WHO Enhanced Gonococcal Antimicrobial Surveillance Programme (EGASP) countries in three WHO regions.

**Methods:**

Isolates from Cambodia (*n* = 474), Indonesia (*n* = 107), Malawi (*n* = 111), the Philippines (*n* = 817), South Africa (*n* = 578), Thailand (*n* = 249), Uganda (*n* = 342) and Vietnam (*n* = 234) were examined. MICs of gepotidacin were determined using agar dilution. Gepotidacin target genes (*gyrA* and *parC*) were examined with Illumina sequencing.

**Results:**

Gepotidacin showed high *in vitro* activity, with MICs ranging from <0.016 to 4 mg/L. The modal MIC was 0.5 mg/L, MIC₅₀ 0.5 mg/L and MIC₉₀ 1 mg/L. Minor variations in the MIC distributions across countries were observed. ParC D86N, which in suboptimal gepotidacin concentrations predisposes for resistance development, was found in 35.5% of isolates.

**Conclusions:**

We show that the *in vitro* susceptibility to gepotidacin in *N. gonorrhoeae* isolates, including 4.3% ceftriaxone-resistant isolates, collected 2021–24 in eight WHO EGASP countries, including five Asian countries, is high. Our findings support gepotidacin’s continued clinical development, registration and introduction as a novel oral treatment for gonorrhoea. However, as with all new novel antimicrobials, cautious and optimal introduction, and surveillance of phenotypic and genomic susceptibility to gepotidacin internationally, pre- and post-licencing, should accompany any clinical implementation.

## Introduction

The global prevalence of gonorrhoea remains alarmingly high, and the treatment of gonorrhoea is increasingly compromised by antimicrobial resistance (AMR) in *Neisseria gonorrhoeae*.^1,2^ The effectiveness of the only remaining empirical first-line therapies, ceftriaxone monotherapy (0.5–1 g) or ceftriaxone (0.5–1 g) combined with azithromycin (1–2 g), is increasingly jeopardized, as resistance to ceftriaxone and especially azithromycin is becoming prevalent internationally.^1–17^ There is an urgent need to develop new antimicrobials, especially oral ones, to ensure effective treatment options for gonorrhoea in the future.

Gepotidacin is a new triazaacenaphthylene bacterial topoisomerase type IIA inhibitor that inhibits DNA replication through a novel mechanism of action that targets both the GyrA subunit of DNA gyrase and the ParC subunit of topoisomerase IV.^18–23^ The binding sites of gepotidacin are different from the ones that fluoroquinolones target in GyrA and ParC, and cross-resistance between gepotidacin and fluoroquinolones, e.g. ciprofloxacin, is lacking.^24–26^ In a recent Phase 3 randomized controlled clinical trial (RCT) evaluating gepotidacin in a dual dose regimen (single oral doses of 3 g plus 3 g 10–12 h later), gepotidacin showed non-inferiority compared with internationally recommended treatment (ceftriaxone 500 mg single intramuscular dose plus azithromycin 1 g single oral dose) for the treatment of uncomplicated urogenital gonorrhoea (ClinicalTrials.gov identifier: NCT04010539).^22^

Gepotidacin has demonstrated high *in vitro* susceptibility among gonococcal isolates, including isolates with ciprofloxacin and multidrug resistance, with MICs of 0.032–4 mg/L and a MIC_90_ of 1 mg/L.^24–26^ However, a pre-existing ParC D86N substitution predisposes for development of gepotidacin resistance as a stepping-stone mutation.^25–28^ When exposed to suboptimal gepotidacin concentrations, e.g. the 3 g monotherapy used in the Phase 2 clinical trial,^20^ gonococcal strains harbouring the ParC D86N substitution can more easily develop a subsequent substitution in the second gepotidacin target GyrA, A92T, which results in a high MIC (≥32 mg/L).^26,27^ It is imperative to examine the susceptibility to gepotidacin and prevalence of substitutions in ParC D86 and GyrA A92 in global gonococcal isolates, particularly isolates with ceftriaxone resistance and from Asian countries, i.e. where the resistance to most antimicrobials has been higher during many decades.^1–3,7,8–10,16,17,29–34^

To monitor global gonococcal AMR, the WHO established the global Gonococcal Antimicrobial Surveillance Programme (GASP).^1,2,29,30^ The WHO has further enhanced, standardized and quality assured the international gonococcal AMR surveillance through the WHO Enhanced GASP (EGASP).^16,32–34^ This includes representative sentinel countries worldwide. As of 2023, the WHO EGASP sentinel countries included Cambodia,^8,10^ Indonesia, Malawi, the Philippines, South Africa, Thailand,^31^ Uganda, Vietnam^9,17^ and Zimbabwe.

This study provides up-to-date international *in vitro* susceptibility data for gepotidacin against clinical gonococcal isolates (*n* = 2912), including 125 (4.3%) ceftriaxone-resistant isolates,^8–10,16,32–34^ collected from 2021 to 2024 in eight WHO EGASP sentinel countries representing WHO South-East Asian Region (Indonesia and Thailand), WHO Western Pacific Region (Cambodia, the Philippines and Vietnam) and WHO African Region (Malawi, South Africa and Uganda).

## Materials and methods

Clinical gonococcal isolates (*n* = 2912; one per gonorrhoea case) from eight countries [Cambodia (*n* = 474), Indonesia (*n* = 107), Malawi (*n* = 111), the Philippines (*n* = 817), South Africa (*n* = 578), Thailand (*n* = 249), Uganda (*n* = 342) and Vietnam (*n* = 234)] collected from 2021 to 2024 through WHO EGASP were examined.^16,32–34^

All isolates were shipped frozen on dry ice to the WHO Collaborating Centre for Gonorrhoea and Other STIs, Sweden, where they were cultured on GCAGP agar medium [3.6% Difco GC Medium Base agar (BD Diagnostics, Sparks, MD, USA) with 1% haemoglobin (BD Diagnostics), 1% IsoVitalex (BD Diagnostics) and 10% horse serum]. Inoculated agar plates were incubated for 20–24 h at 36 ± 1°C in a humid CO_2_-enriched atmosphere. In cases of uncertain colony morphology or MIC results, isolates were re-verified as *N. gonorrhoeae* using MALDI-TOF MS (Bruker Daltonics, Bremen, Germany). The MIC (mg/L) of gepotidacin was determined by agar dilution technique, according to CLSI guidelines (www.clsi.org) on GCVIT agar plates [3.6% Difco GC Medium Base agar (BD Diagnostics) supplemented with 1% IsoVitalex (BD Diagnostics)]. The examined gepotidacin MICs ranged from 0.016 to 8 mg/L, and two plates lacking gepotidacin were included for quality control. The 2024 WHO reference strains F and L^35^ and ATCC 49 226 were used for quality control of each testing batch. Oxidase testing was used to resolve uncertainty regarding growth. The GyrA A92T and ParC D86N substitutions, associated with gepotidacin resistance when found together,^25–27^ were identified using whole-genome sequencing, as previously described.^4^

## Results and discussion

The results of the gepotidacin susceptibility testing of the 2912 clinical gonococcal isolates collected from 2021 to 2024 in eight WHO EGASP countries are summarized in Table 1.

**Table 1.** Susceptibility to gepotidacin^a^ in clinical *N. gonorrhoeae* isolates (*n* = 2912) collected from 2021 to 2024 in eight WHO EGASP countries in three WHO regions

Country	Years of isolation	Number of isolates	Modal MIC (mg/L)	MIC range (mg/L)	MIC_50_ (mg/L)	MIC_90_ (mg/L)
Cambodia	2021	11	1	0.25–2	1	2
	2022	145	0.5	<0.016–2	0.5	2
	2023	253	0.5	0.032–4	0.5	1
	2024	65	0.5	0.064–2	0.5	1
Indonesia	2023	107	0.25	0.064–2	0.25	0.5
Malawi	2023	111	0.25	0.032–1	0.25	1
The Philippines	2022	245	0.5	<0.016–4	0.5	1
	2023	572	0.5	0.064–2	0.5	1
South Africa	2022	287	0.25	0.064–2	0.25	1
	2023	291	0.25	<0.016–2	0.25	1
Thailand	2022	99	0.5	0.125–2	0.5	1
	2023	150	0.5	0.064–2	0.5	1
Uganda	2022	136	0.25	0.064–1	0.25	0.5
	2023	206	0.25	0.064–1	0.5	1
Vietnam	2023	234	0.5	0.032–4	0.5	2
Total		2912	0.5	<0.016–4	0.5	1

WHO, World Health Organization; EGASP, Enhanced Gonococcal Antimicrobial Susceptibility Programme; MIC_50_, concentration where 50% of isolates inhibited; MIC_90_, concentration where 90% of isolates inhibited.

^a^Gepotidacin MICs (mg/L) were determined by agar dilution method in accordance with recommendations by the CLSI (www.clsi.org).

The MICs of gepotidacin ranged from <0.016 to 4 mg/L. Three (0.1%) isolates had the lowest MIC value (<0.016 mg/L). The highest MIC value (4 mg/L) was detected in five (0.2%) isolates, from Cambodia (*n* = 1), the Philippines (*n* = 2) and Vietnam (*n* = 2), and 138 (4.7%) isolates had a MIC of 2 mg/L. Modal MIC for all isolates was 0.5 mg/L, varying from 0.25 mg/L (isolates from Indonesia 2023, Malawi 2023, South Africa 2022 and 2023 and Uganda 2022 and 2023) to 1 mg/L (Cambodia 2021). Overall MIC_50_ was 0.5 mg/L, varying from 0.25 mg/L (Indonesia 2023, Malawi 2023, South Africa 2022 and 2023 and Uganda 2022) to 1 mg/L (Cambodia 2021). Overall MIC_90_ was 1 mg/L, ranging from 0.5 mg/L (Indonesia and Uganda 2022) to 2 mg/L (Cambodia 2022 and 2023 and Vietnam 2023) (Table 1).

**Figure 1.**
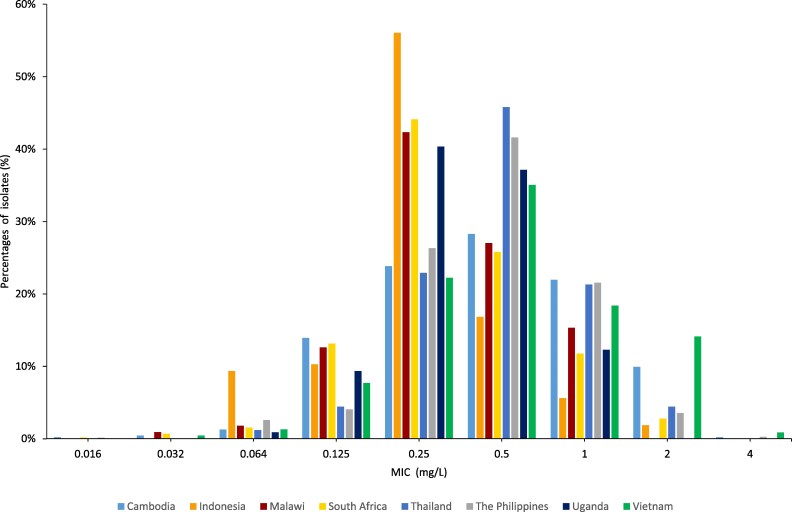
MIC distributions for gepotidacin in *N. gonorrhoeae* isolates (*n* = 2912) from eight WHO EGASP countries in three WHO regions, 2021 (*n* = 11), 2022 (*n* = 912), 2023 (*n* = 1924) and 2024 (*n* = 65).

The WHO EGASP isolates mainly represented the previously described gepotidacin wild-type MIC distribution,^24–26^ but five isolates had an MIC of 4 mg/L (Figure 1). Using ECOFFinder (https://clsi.org/resources/ecoffinder), the fitted wild-type MIC distribution resulted in an ECOFF99 at 2 mg/L (ECOFF95 = 1 mg/L; ECOFF97.5/99/99.5 = 2 mg/L; ECOFF99.9 = 4 mg/L). Notably, an ECOFF at 2 mg/L has recently been suggested by EUCAST (https://mic.eucast.org/search/diagram/56777). The MIC distributions from Cambodia, Thailand, the Philippines and Vietnam appeared to have slightly shifted towards higher MIC values. Isolates from Vietnam were also overrepresented among the higher MIC values with 14% (33/234) of the isolates having an MIC of 2 mg/L and 1% (3/234) having an MIC of 4 mg/L (Figure 1). No GyrA A92T^26,27^ substitution was found. However, the ParC D86N substitution,^4,25–27^ which in suboptimal concentrations of gepotidacin predisposes for development of gepotidacin resistance, was found in 35.5% (1035/2912) of isolates. Notably, the prevalence of ParC D86N widely varied across countries, from 1.8% (Malawi) to 50.9% (the Philippines). The modal gepotidacin MIC in isolates with ParC D86N was 0.5 mg/L, compared to 0.25 mg/L in isolates with ParC D86 wild type. Furthermore, 62 (44.9%) and 2 (40.0%) of the isolates with gepotidacin MIC of 2 mg/L and 4 mg/L, respectively, had the ParC D86N substitution. Promisingly, ParC D86N was less common among ceftriaxone-resistant isolates (13.8%) than ceftriaxone-susceptible isolates (37.8%).

The present study examined 2912 isolates collected from 2021 to 2024 in eight WHO EGASP countries in the WHO South-East Asian Region (Indonesia and Thailand), WHO Western Pacific Region (Cambodia, the Philippines and Vietnam) and WHO African Region (Malawi, South Africa and Uganda) for their susceptibility to gepotidacin. Gepotidacin, the first-in-class triazaacenaphthylene, recently showed non-inferiority to internationally recommended treatment ceftriaxone plus azithromycin in the treatment of uncomplicated urogenital gonorrhoea.^22^ The present study shows a high *in vitro* susceptibility to gepotidacin in a large and broad international collection of gonococcal isolates. The material comprised a wide range of resistance including resistance to ceftriaxone (4%) and azithromycin (4%).^8–10,16,32–34^ Nevertheless, it is a concern that the ParC D86N substitution was identified in 35.5% of isolates, which is substantially higher than the previously reported 11.2% of gonococcal isolates in Europe.^4^ It has been previously shown that gonococcal strains with ParC D86 substitution that are exposed to suboptimal concentrations of gepotidacin can select a GyrA A92T substitution resulting in high gepotidacin MIC values (>32 mg/L).^26,27^ Worryingly, 44.9% and 40.0% of the isolates with the highest gepotidacin MICs (2 mg/L and 4 mg/L, respectively) had the ParC D86N stepping-stone substitution. Furthermore, in March 2025, gepotidacin was approved for the treatment of uncomplicated urinary tract infection in female adults and paediatric patients 12 years of age and older (www.gsk.com/en-gb/media/press-releases/blujepa-gepotidacin-approved-by-us-fda-for-treatment-of-uncomplicated-urinary-tract-infections), which may increase the selective pressure for development of resistance also in *N. gonorrhoeae* in subjects with co-infections.^36^ In August 2025, gepotidacin was accepted for priority review by the US FDA for the oral treatment of uncomplicated urogenital gonorrhoea (https://us.gsk.com/media/0qea12sa/gepo-gc-us-filing-acceptance_sea_final.pdf).

The limitations of this study are inherent limitations in WHO EGASP and include the limited number of participating sentinel countries and gonococcal isolates examined and inclusion of only urethral discharge specimens from males.

In conclusion, the present study shows a high *in vitro* activity of gepotidacin against contemporary *N. gonorrhoeae* isolates, including 4.3% (*n* = 125) ceftriaxone-resistant isolates,^8,10,16,32–34^ collected from 2021 to 2024 in eight WHO EGASP countries, including five Asian countries. Our results support further development, registration and introduction of gepotidacin as an alternative treatment for uncomplicated gonorrhoea. However, as with all new novel antimicrobials, it is imperative with cautious and optimal introduction,^36,37^ and to survey the susceptibility of gepotidacin, phenotypically and genome-based, internationally, i.e. pre-licencing and post-licencing. This will be essential to effectively identify any emergence or transmission of gepotidacin resistance.

## WHO EGASP Gepotidacin Study Group

Cambodia: Lon Say Heng, Vichea Ouk, Mot Virak, Phal Kun Mom and Serongkea Deng; Indonesia: Vivi Setiawaty, Endang Lukitosari, Nurhalina Afriana, Verawati Sulaiman, Teguh Hartono, Maria Laurensia and Ni Luh Putu Pitawati; Malawi: Mitch Matoga, Irving Hoffman and Robert Krysiak; the Philippines: Sonia Sia, Manuel C Jamoralin Jr., Marietta Lagrada, June Gayeta and Jaywardeen Abad; South Africa: Venessa Maseko, Etienne Müller, Lindy Gumede and Duduzile Valashiya; Thailand: Rossaphorn Kittiyaowamarn, Natnaree Girdthep, Porntip Paopang, Pongsathorn Sangprasert, Thitima Cherdtrakulkiat and Jaray Tongtoyai; Uganda: Francis Kakooza, Peter Kyambadde, Emmanuel Mande, Martha Nakasi and Dickson Tabajjwa; Vietnam: Le Huu Doanh, Pham Thi Lan, Pham Quynh Hoa, Pham Dieu Hoa, Thuy Thi Phan Nguyen, Hao Trong Nguyen, Nhi Thi Uyen Pham and Phuong Thi Thanh Nguyen; WHO Vietnam: Nguyen Thi Thuy Van; WHO CC-Australia: Monica Lahra; WHO HQ: Teodora Wi, Ismael Maatouk and Phiona Vumbugwa; WHO CC-Sweden: Magnus Unemo, Daniel Golparian, Susanne Jacobsson and Daniel Schröder.
